# Editorial Department

**Published:** 1856-03

**Authors:** 


Messrs. A. I. Mathews & Co., of Buffalo, whose name is so familiar to every one who takes our Journal, abandons his old stand on the 4th of March, and opens a new store on the west side of Main street, (No. 220) directly opposite his former location.

Mr. Mathews has, by selling good drugs and devoting himself to the profession to the exclusion of patent medicines, putty and paints, been enabled to fit up his new quarters at an expense of some $10,000, and has by far the handsomest and most convenient drug store we ever saw. It is probably not equalled in any city for beauty, neatness and convenience.

Prof. Hadley continues to have charge of the manufacturing department, and has a commodious laboratory above the store.

We are glad to notice this marked evidence of prosperity on the part of this firm. They have richly earned it by probity, industry, and close attention to the details of their business.

See advertisement on fourth page of cover.

